# Targeted Sequencing-Based Re-Evaluation of Candidate Immunogenetic Variants for Biologic Treatment Response and Difficult-to-Treat Rheumatoid Arthritis

**DOI:** 10.3390/ijms27198800

**Published:** 2026-10-01

**Authors:** Alena I. Zagrebneva, Elena N. Simonova, Yuliya A. Gavrikova, Vladislav V. Dolgov, Anastasiia A. Buianova, Ekaterina D. Glumova, Maria I. Tubalova, Galina S. Koksharova, Artem R. Nurislamov, Vadim I. Mazurov

**Affiliations:** 1 Organizational and Methodological Department, North-Western State Medical University Named After I.I. Mechnikov, 41, Kirochnaya St., Saint Petersburg 191015, Russia; maz.nwgmu@yandex.ru; 2 Moscow Clinical Research Center “Hospital No. 52”, 3, Pekhotnaya St., Moscow 123182, Russia; 4130524@mail.ru (E.N.S.); jgavrikovagkb52@gmail.com (Y.A.G.); drdolgovvv@gmail.com (V.V.D.); anastasiiabuianova97@gmail.com (A.A.B.); 3 Research Institute of Healthcare Organization and Medical Management, Moscow Department of Healthcare, 30, Bolshaya Tatarskaya St., Moscow 115184, Russia; 4 Russian Clinical Research Center of Gerontology, Pirogov Russian National Research Medical University of the Ministry of Healthcare of the Russian Federation, Moscow 129226, Russia; 5 Institute of Cytology and Genetics, Russian Academy of Sciences, Novosibirsk 630090, Russia; glumova-katerinka@mail.ru (E.D.G.); m.tubalova@g.nsu.ru (M.I.T.); g.koksharova@alumni.nsu.ru (G.S.K.); a.nurislamov@alumni.nsu.ru (A.R.N.); 6 Clinical Rheumatology Hospital No. 25 Named After V.A. Nasonova, 30, Bolshaya Podyacheskaya St., Saint Petersburg 190068, Russia

**Keywords:** rheumatoid arthritis, pharmacogenetics, biologic DMARDs, TNF-α inhibitors, IL-6 inhibitors, difficult-to-treat RA, candidate-variant analysis, targeted sequencing

## Abstract

Genetic determinants of response to biologic disease-modifying antirheumatic drugs (bDMARDs) in rheumatoid arthritis (RA) remain insufficiently characterized. We developed a targeted sequencing panel of candidate immunogenetic variants identified through a literature review and evaluated associations with response to TNF-α inhibitors, response to IL-6 inhibitors, and difficult-to-treat RA (D2TRA). Whole-blood DNA was sequenced using custom hybridization capture on a DNBSEQ-G400 platform (2 × 150 bp). Reads were aligned to hg38 using BWA-MEM, and variants were called with GATK HaplotypeCaller. After quality control, the D2TRA analysis included 154 patients (59 D2TRA and 95 non-D2TRA); drug-response analyses included 66 anti-TNF patients (26 responders, 40 non-responders) and 69 anti-IL-6 patients (29 responders, 40 non-responders). Treatment-response associations were assessed using unadjusted allelic chi-square tests with multiple-testing correction; D2TRA was analyzed using logistic regression adjusted for age, sex, and body mass index. For IL-6 inhibitor response, rs11656130/*MAP2K6* (*p* = 0.0163; FDR = 0.3704) and rs4910008/*GALNT18* (*p* = 0.0212; FDR = 0.3704) were identified, while rs7767069/*LINC02549* was associated with TNF-α inhibitor response (*p* = 0.0171; FDR = 0.5968). For D2TRA, rs1813443 in *CNTN5* (OR = 1.85, *p* = 0.017) and rs12081765, an intergenic variant near *LMX1A*/*RXRG* (OR = 0.59, *p* = 0.028), were nominally associated; neither association remained significant after multiple-testing correction. With the current sample sizes, the study could detect only large genetic effects, and was underpowered for these after Bonferroni correction (α = 0.00143). The findings highlight the limited reproducibility of candidate pharmacogenetic markers in RA and provide a targeted panel and analytical framework for larger replication studies.

## 1. Introduction

Rheumatoid arthritis (RA) is a chronic systemic autoimmune disease characterized by persistent synovial inflammation, progressive joint damage, and substantial functional impairment. Despite major advances in the understanding of RA pathogenesis and the development of disease-modifying antirheumatic drugs (DMARDs), complete and sustained disease control remains difficult to achieve in a proportion of patients [1]. Contemporary genetic studies have further demonstrated that RA is a highly heritable and genetically heterogeneous disease, with numerous susceptibility loci implicating immune regulation and joint tissue biology [2]. The clinical and biological heterogeneity of RA is also reflected in substantial interindividual variability in response to targeted therapies [1].

Current treatment strategies follow a treat-to-target approach, beginning with conventional synthetic DMARDs (csDMARDs), particularly methotrexate (MTX), and progressing to biological or targeted synthetic DMARDs (bDMARDs/tsDMARDs) when the treatment target is not achieved [1]. The 2025 update of the European Alliance of Associations for Rheumatology (EULAR) recommendations recommends initial treatment with MTX, preferably in combination with short-term glucocorticoids, followed, in patients with an insufficient response after 3–6 months, by addition of a bDMARD; a Janus kinase inhibitor (JAKi) may also be considered after careful assessment of relevant safety factors. If the first bDMARD or JAKi fails, treatment with another bDMARD or JAKi is recommended [3]. Thus, contemporary RA management provides multiple therapeutic options, but treatment selection remains largely based on sequential assessment of clinical response rather than validated molecular predictors of efficacy.

A particularly challenging clinical phenotype is difficult-to-treat RA (D2TRA). The EULAR definition was developed to establish a uniform framework for patients who remain symptomatic despite treatment according to current recommendations [4]. D2TRA requires failure of at least two bDMARDs or tsDMARDs with different mechanisms of action after csDMARD therapy, together with evidence of persistent or progressive disease or disease-related symptoms and a perception by the patient and/or rheumatologist that disease management is problematic [4]. Importantly, D2TRA should not be equated with pharmacological resistance. The syndrome encompasses heterogeneous mechanisms, including persistent inflammatory activity, comorbidities, treatment-related factors, and conditions that may mimic or amplify disease activity [5,6,7]. Diagnostic assessment is therefore an essential component of D2TRA management, as pain, fibromyalgia, obesity, structural damage, and other comorbid conditions may contribute to persistent symptoms without necessarily reflecting ongoing synovial inflammation [6].

The clinical relevance of this phenotype is supported by population-based data. In a large RA registry, Paudel et al. reported that approximately 5–20% of patients with RA may fail multiple treatments and meet the broader concept of difficult-to-treat disease; application of the EULAR definition identified a clinically distinct subgroup with greater disease burden and treatment complexity [8]. More recent work emphasizes that D2TRA represents a heterogeneous disease state in which biological factors coexist with comorbidities, altered pain processing, and other determinants of persistent symptoms [7]. Consequently, treatment failure in D2TRA cannot be attributed to a single mechanism, and the identification of biological determinants that distinguish therapeutic response remains an important unmet need.

Tumor necrosis factor-α (TNF-α) and interleukin-6 (IL-6) are among the principal inflammatory pathways targeted by biologic therapy in RA, making genetic variation within these pathways a biologically plausible source of interindividual differences in treatment response [9]. Candidate-gene studies have investigated the *TNF-α* −308 G/A polymorphism as a potential predictor of response to TNF-α blockade. O’Rielly et al. reported an association between the −308A allele and poorer response to TNF-α inhibitors in a systematic review and meta-analysis [10]. However, the subsequent meta-analysis by Lee et al. found no overall association between the −308 polymorphism and response to TNF blockers, although an association between the *TNF-α* −238 polymorphism and response to infliximab was observed after stratification by individual TNF inhibitor [11]. Maxwell et al. further demonstrated that the *TNF-α* −308 variant could be associated with differential response to individual anti-TNF agents, suggesting that any effect of this locus may be treatment-specific rather than a universal predictor of TNF blockade efficacy [12]. These findings illustrate both the biological plausibility and the inconsistent reproducibility of candidate pharmacogenetic associations in RA.

Genetic predictors of response to IL-6 pathway inhibition have likewise been investigated. Maldonado-Montoro et al. identified associations between selected *IL6R* polymorphisms and clinical response measures during tocilizumab treatment, although the same study also demonstrated the importance of clinical treatment history and baseline disease activity as determinants of outcome [13]. Genome-wide analyses have provided additional evidence that response to tocilizumab may involve multiple genetic loci rather than a single dominant variant [14]. Similarly, an IL-6 pathway-driven investigation integrating genotype, transcript, and protein measurements identified biological heterogeneity within the IL-6 pathway in relation to disease activity and response to tocilizumab [15]. These observations support the investigation of pathway-level genetic variation while also indicating that treatment response is unlikely to be explained by individual variants alone.

Importantly, genetic variation represents only one component of the biological heterogeneity underlying treatment response. Genome-wide association studies (GWAS) have identified multiple loci associated with RA susceptibility and immune regulation [2], whereas studies of treatment response suggest that molecular characteristics of the target tissue may also influence therapeutic efficacy. For example, Nerviani et al. demonstrated that a pauci-immune synovial pathotype was associated with an inadequate response to TNF-α blockade, supporting the concept that clinically similar patients may have distinct underlying biological states that influence response to the same therapeutic mechanism [16]. Thus, the search for pharmacogenetic predictors should be considered within a broader framework of molecular heterogeneity rather than as an isolated explanation for treatment failure.

Taken together, the available evidence provides a rationale for evaluating previously reported candidate variants while also highlighting the limitations of individual-marker approaches. The inconsistency of associations across cohorts, therapeutic agents, and clinical response definitions, together with the multifactorial nature of D2TRA, makes independent evaluation of candidate pharmacogenetic markers particularly important. In this context, we assessed a literature-derived targeted sequencing panel of immunogenetic variants previously implicated in response to TNF-α and IL-6 pathway inhibition [10,11,12,13,14,15,17,18]. Associations were evaluated across three clinically defined phenotypes: response to TNF-α inhibitors, response to IL-6 inhibitors, and D2TRA status. We aimed to determine whether previously reported candidate variants were reproducibly associated with biologic treatment response or with the difficult-to-treat phenotype in RA.

## 2. Results

### 2.1. Clinical and Demographic Characteristics

A total of 194 patients with RA receiving b/tsDMARDs were analyzed: 96 (49.5%) fulfilled D2TRA criteria, and 98 (50.5%) did not. The groups were comparable in age (58.3 [50.3; 65.5] vs. 61.8 [49.2; 66.9] years; *p* = 0.371), sex (females 87.5% vs. 84.7%; *p* = 0.720), height (165.0 [162.0; 172.0] vs. 166.0 [162.0; 170.0] cm; *p* = 0.862), weight (73.0 [62.0; 86.0] vs. 73.0 [62.5; 85.0] kg; *p* = 0.908), disease duration from onset (13.6 [9.7; 20.0] vs. 13.4 [7.9; 19.9] years; *p* = 0.660) and from verification, and age at disease onset (44.9 [36.6; 50.7] vs. 44.8 [34.2; 55.3] years; *p* = 0.583). No differences were found in seropositivity for rheumatoid factor (RF) (*p* = 0.828), anti-citrullinated protein antibodies (ACPA) (*p* = 0.353), or antinuclear antibodies (ANA) (*p* = 0.860).

Patients with D2TRA had significantly higher disease activity: Disease Activity Score 28-C-reactive protein (DAS28-CRP) 3.37 [2.21; 4.58] vs. 2.20 [1.78; 2.86] (*p* < 0.001), DAS28-erythrocyte sedimentation rate (DAS28-ESR) 3.78 [2.68; 4.95] vs. 2.46 [1.88; 3.04] (*p* < 0.001), Clinical Disease Activity Index (CDAI) 14.0 [6.0; 21.0] vs. 6.0 [4.0; 8.0] (*p* < 0.001), and Simplified Disease Activity Index (SDAI) 14.4 [6.6; 22.8] vs. 6.2 [4.3; 9.0] (*p* < 0.001). Morning stiffness, 28-joint tender joint count (TJC-28), and 28-joint swollen joint count (SJC-28) were also significantly higher in D2TRA (all *p* < 0.001). Visual analogue scale (VAS) scores were higher for both patient (5.0 [3.0; 6.0] vs. 3.0 [2.0; 4.0]) and physician (3.0 [2.0; 5.0] vs. 2.0 [2.0; 3.0]) assessments (both *p* < 0.001). The distribution of functional class differed significantly (*p* < 0.001): class III–IV was observed in 31.3% of D2TRA vs. 10.2% of non-D2TRA. Among laboratory parameters, D2TRA was associated with higher ESR (23.0 vs. 12.0 mm/h; *p* = 0.001), higher white blood cell (WBC) (6.32 vs. 5.43 × 10^9^/L; *p* < 0.001), lower IgG (9.96 vs. 11.20 g/L; *p* = 0.005), lower 25(OH)D_3_ (32.3 vs. 38.0 ng/mL; *p* = 0.017), and altered lymphocyte subsets (all *p* < 0.05).

Quality of life was significantly lower in the D2TRA group across all eight the Short Form-36 (SF-36) domains: physical functioning (30.0 vs. 50.0), role-physical (0 vs. 25.0), pain (45.0 vs. 57.0), general health (45.0 [30.0; 50.0] vs. 45.0 [35.0; 60.0]), mental health (58.0 vs. 68.0), role-emotional (33.0 vs. 99.0), social functioning (56.0 vs. 75.0), and vitality (40.0 vs. 55.0) (all *p* < 0.05). Median Health Assessment Questionnaire (HAQ) was 1.75 [1.13; 2.00] in D2TRA vs. 0.88 [0.38; 1.50] in non-D2TRA (*p* < 0.001); EuroQol five-dimension five-level questionnaire (EQ-5D-5L) score was 55.0 [40.0; 70.0] vs. 70.0 [50.0; 80.0] (*p* = 0.001). On Personality Inventory for DSM-5—Brief Form (PID-5-BF), significant differences were observed for negative affectivity (1.23 vs. 0.83; *p* = 0.005), antagonism (0.70 vs. 0.33; *p* = 0.009), disinhibition (1.00 vs. 0.67; *p* = 0.029), anancastia (1.50 vs. 0.67; *p* < 0.001), and psychoticism (0.70 vs. 0.33; *p* = 0.019), whereas detachment did not differ (*p* = 0.268). Functional Assessment of Chronic Illness Therapy (FACIT) was lower in D2TRA (26.0 vs. 31.0; *p* = 0.008), Fibromyalgia Rapid Screening Tool 2010 (FIRST2010) higher (4.0 vs. 2.0; *p* < 0.001). Patient Health Questionnaire-9 (PHQ-9) showed a borderline difference (6.0 vs. 4.0; *p* = 0.059).

### 2.2. Treatment Response: Clinical and Quality-of-Life Characteristics

Among 66 patients treated with anti-TNF, 26 responded, and 40 did not. Responders had significantly lower disease activity (DAS28-CRP 2.27 [1.78; 3.04] vs. 3.33 [2.39; 4.32]; *p* = 0.001; CDAI 6.0 [4.0; 10.0] vs. 14.0 [7.0; 19.3]; *p* < 0.001; SDAI 7.1 [4.9; 10.1] vs. 15.2 [7.5; 20.6]; *p* < 0.001), lower morning stiffness (1.0 vs. 4.0; *p* = 0.018), lower TJC-28 (1.0 vs. 3.0; *p* = 0.011) and SJC-28 (0.0 [0.0; 0.0] vs. 0.0 [0.0; 2.0]; *p* = 0.012), and lower VAS scores (patient 3.0 vs. 5.0; physician 2.0 vs. 3.5; both *p* ≤ 0.001), and they less frequently had wrist erosions (69.2% vs. 92.5%; *p* = 0.019). Quality of life was significantly better in responders: HAQ 0.63 [0.16; 1.57] vs. 2.00 [1.44; 2.00] (*p* < 0.001); SF-36 physical functioning 55.0 vs. 25.0 (*p* = 0.008); pain 67.0 vs. 45.0 (*p* < 0.001); role-emotional 100 vs. 33 (*p* = 0.048); FIRST2010 1.0 vs. 4.0 (*p* < 0.001); EQ-5D-5L score 75.0 vs. 59.5 (*p* = 0.072).

Among 69 patients treated with anti-IL6, 29 responded, and 40 did not. Responders had lower disease activity (DAS28-CRP 1.78 [1.51; 2.30] vs. 3.04 [2.20; 4.15]; *p* < 0.001; CDAI 5.0 [4.0; 6.0] vs. 12.5 [6.75; 19.0]; *p* < 0.001; SDAI 5.2 [4.0; 6.4] vs. 12.9 [6.9; 19.7]; *p* < 0.001), lower fibrinogen (2.64 [2.27; 3.14] vs. 3.41 [2.54; 4.05] g/L; *p* = 0.014), lower TJC-28 (*p* < 0.001) and SJC-28 (*p* = 0.020), and lower VAS scores (both *p* ≤ 0.004), and they less frequently had advanced functional class (class III: 3.4% vs. 35.0%; *p* = 0.005). Age at disease onset was younger in responders (36.2 [27.5; 50.7] vs. 46.8 [39.0; 53.4] years; *p* = 0.0501), although this difference was borderline. Quality of life: HAQ 1.19 [0.38; 1.47] vs. 1.81 [1.03; 2.00] (*p* = 0.006); EQ-5D-5L score 72.5 vs. 55.0 (*p* = 0.015); SF-36 physical functioning 50.0 vs. 30.0 (*p* = 0.009); pain 51.5 vs. 45.0 (*p* = 0.030); FIRST2010 2.0 vs. 4.0 (*p* = 0.013).

### 2.3. Association Analysis: Anti-IL6 and Anti-TNF Response (Unadjusted Screen)

For anti-IL6 and anti-TNF response, the distribution of unadjusted *p*-values did not show an obvious excess of small *p*-values (median unadjusted *p* ≈ 0.46–0.55, depending on phenotype) (Table 1). No variant remained statistically significant for either phenotype after correction for multiple comparisons (all corrected *p* > 0.05 by Bonferroni and FDR methods).

No other variant reached nominal significance for either phenotype; the minimum FDR-adjusted *p*-values were approximately 0.37–0.60, and the minimum Bonferroni-adjusted *p*-values approximately 0.57–0.74, indicating that the most promising uncorrected signals were far from surviving correction for the 35 variants tested per phenotype.

### 2.4. Association Analysis: D2TRA (Primary, Covariate-Adjusted)

**Figure 1:**
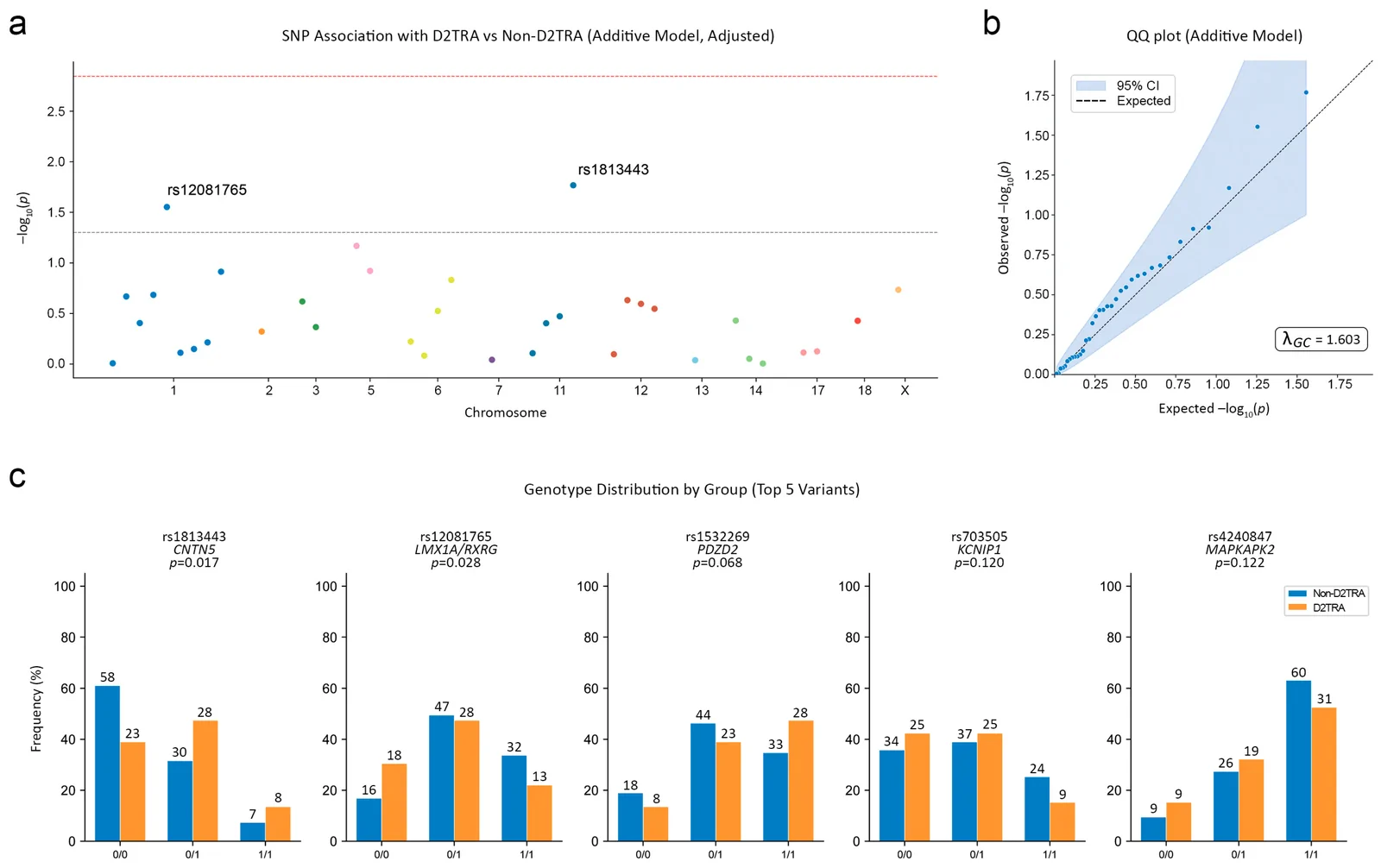
Association results from the D2TRA covariate-adjusted additive logistic regression model (154 patients: 59 D2TRA, 95 non-D2TRA; 35 variants). (**a**) Manhattan-style plot of unadjusted −log_10_(*p*) values by genomic position. Dashed gray line: nominal *p* = 0.05; dashed red line: Bonferroni-corrected threshold (α/35 = 0.00143). (**b**) Quantile–quantile (QQ) plot of observed versus expected −log_10_(*p*) values under the null. Genomic inflation factor λ_GC_ = 1.60. (**c**) Genotype frequency distributions for the top 5 variants ranked by unadjusted *p*-value. Blue, non-D2TRA patients; orange, D2TRA patients. Numbers above bars indicate raw genotype counts.

In the 154-patient D2TRA analysis set (59 D2TRA, 95 non-D2TRA), the additive logistic regression model adjusted for age, sex, and body mass index identified two variants at nominal significance: rs1813443 (*CNTN5*; OR = 1.85, *p* = 0.017), associated with increased odds of D2TRA status, and rs12081765 (intergenic, near *LMX1A*/*RXRG*; OR = 0.59, *p* = 0.028), associated with decreased odds (Figure 1a) (Table 2). Neither variant survived FDR correction (minimum FDR-adjusted *p* = 0.49) or Bonferroni correction. Unadjusted Cochran–Armitage trend and Fisher’s exact sensitivity analyses were concordant with the adjusted results for both variants.

The estimated genomic inflation factor for this model was λ_GC_ = 1.60 (Figure 1b); given the small number of tested variants (35) and the candidate-based, non-genome-wide design of this panel, this metric should be interpreted cautiously and cannot be used to distinguish population stratification from small-sample variability.

Genotype counts for the top-ranked variants, including rs1813443 and rs12081765, are shown in Figure 1c.

## 3. Discussion

This study evaluated a literature-derived panel of 35 candidate immunogenetic variants, previously implicated in response to TNF-α inhibition, IL-6 inhibition, or in D2TRA susceptibility [4]. After correction for multiple testing, no variant showed a statistically significant association with any of the three phenotypes examined: response to anti-TNF agents, response to anti-IL-6 agents, or D2TRA status. These negative findings should be interpreted in the context of the limited statistical power of the present cohort, rather than as evidence against the reproducibility of the tested variants. The study was underpowered to detect modest effects at the Bonferroni-corrected threshold, and a drug-specific or time-dependent effect could have been missed. More broadly, the field has struggled to identify robust, clinically actionable predictors from isolated candidate-gene studies in small-to-moderate-sized cohorts, and this remains an unresolved challenge that will require adequately powered, harmonized, multi-center studies.

### 3.1. Contextualizing the Anti-TNF and Anti-IL-6 Findings

The most extensively studied pharmacogenetic marker in RA is the *TNF-α* −308 G/A polymorphism (rs1800629) [10]. Initial reports suggested that the −308 A allele was associated with poorer response to TNF-α blockers, but subsequent meta-analyses yielded conflicting results, including a null finding in a large meta-analysis of 2127 patients [11]. Maxwell et al. further demonstrated that the association with rs1800629 was present for etanercept but not for infliximab [12], suggesting a drug-specific rather than class-wide effect. This pattern of inconsistency—varying by cohort, drug, response definition, and genetic model—has been a recurring theme for many of the variants included in our panel. The absence of a significant association in our cohort is consistent with the broader literature, although the limited power of the present study precludes any conclusion about the reproducibility of these variants.

Importantly, in our cohort, patients received all types of TNF-α inhibitors (adalimumab, etanercept, golimumab, certolizumab pegol) and all types of IL-6 inhibitors (tocilizumab, levilimab, olokizumab, sarilumab) available in clinical practice. Sequential switches between agents were driven by individual clinical course, comorbidities, and infection risks, and the choice of agent was determined by a rheumatology commission/consilium rather than by a pre-specified protocol. This real-world treatment pattern introduces an additional source of heterogeneity: if a genetic effect is drug-specific (as has been shown for rs1800629 with etanercept but not infliximab), pooling all agents within a class may reduce statistical power and potentially mask a true association.

The same sources of heterogeneity—differences in response definition, timing of assessment, and specific drug—have been repeatedly cited as explanations for the poor reproducibility of candidate-gene findings in RA. We therefore interpret our negative results in the context of these methodological limitations and do not exclude the possibility that a drug-specific or time-dependent genetic effect could have been missed.

For IL-6 pathway inhibition, the most frequently studied marker is the *IL6R* rs12083537 polymorphism. This variant has since emerged as one of the more consistent genetic predictors of tocilizumab response [13]. In our cohort, none of the *IL6R* variants tested showed a significant association with anti-IL-6 response after correction for multiple testing (minimum corrected *p* > 0.05 for all). This is consistent with the hypothesis that any genetic effect on IL-6 pathway response is likely to be modest in magnitude and may be modulated by clinical covariates such as disease duration, baseline activity, prior treatment history, and concomitant medications—factors that were not adjusted for in our unadjusted allelic screen.

### 3.2. D2TRA: A Genetically Complex and Heterogeneous Phenotype

The D2TRA phenotype, as defined by the 2021 EULAR criteria [4], represents a particularly challenging endpoint for genetic association studies. Unlike binary response to a single drug class, D2TRA is a composite phenotype that captures failure of at least two b/tsDMARDs with different mechanisms of action, persistent disease activity or progression, and perception of disease management as problematic by the physician and/or patient. Importantly, D2TRA is not synonymous with pharmacological resistance; it encompasses multiple clinical and biological subtypes, including persistent inflammatory refractory RA (PIRRA), non-inflammatory refractory RA (NIRRA), and cases driven primarily by comorbidities, chronic pain, or low treatment adherence [7,19].

It is estimated that approximately half of patients with D2TRA exhibit evidence of persistent inflammatory activity, while the other half have predominantly non-inflammatory mechanisms [19]. The latter group—NIRRA—has been linked to the fibroid/pauci-immune synovial pathotype and is characterized by less severe synovitis, fewer lymphoid aggregates, and lower immune cell markers compared with PIRRA [6,20]. Standard RA-associated variants (*HLA-DRB1*, *PTPN22*) predict disease risk but do not distinguish these subtypes, and a risk score incorporating 76 RA risk single nucleotide polymorphisms (SNPs) and 4 *HLA-DRB1* amino acid positions failed to predict response to first-line TNF inhibitors [19,21,22]. This is consistent with the broader observation that even large GWAS of anti-TNF response have not identified robust predictors of treatment outcome [17]. To date, no direct evidence has demonstrated a causal link between particular (epi)genetic factors and the development of D2T disease [19]. The most relevant direction is probably the genetics of chronic pain and central sensitization, including candidate pain-related genes such as *COMT*, *OPRM1*, *HTR2A*, and *SCN9A*, as well as polygenic risk scores (PRS) for fibromyalgia, which has been shown to influence pain perception independently of inflammation [23,24], and transcriptomic analyses of peripheral blood mononuclear cells (PBMCs) in D2TRA have identified neuroinflammatory signatures, including alterations in NRG1, NEGR1, and S100B [19,25]. However, no validated genetic test can currently distinguish inflammatory from pain-predominant D2TRA in an individual patient, and the genetic architectures of pain and inflammation susceptibility partially overlap, suggesting a continuum rather than two discrete categories [19].

This heterogeneity has profound implications for genetic association studies. If D2TRA comprises several distinct endotypes with different underlying pathophysiological mechanisms—some inflammatory, others non-inflammatory—then pooling all D2TRA patients into a single case group may dilute any genuine genetic signal that is specific to one subtype. Moreover, the D2TRA phenotype is itself dynamic: a substantial proportion of patients who meet D2TRA criteria at one time point may no longer do so after treatment optimization or resolution of confounding factors. This temporal instability further complicates genetic association analyses and may contribute to the lack of robust, reproducible genetic markers for D2TRA [7].

Patients with D2TRA in our cohort exhibited significantly higher disease activity, worse functional status, and markedly reduced quality of life compared with non-D2TRA patients. These observations are consistent with the concept of D2TRA as a heterogeneous state that encompasses both a genuinely inflammatory component and a non-inflammatory, symptom-driven component [7,19]. The reduction across all SF-36 domains, elevated HAQ scores (1.75 vs. 0.88; *p* < 0.001), and lower EQ-5D-5L scores (55.0 vs. 70.0; *p* = 0.001) observed in our D2TRA group underscore the substantial psychosocial burden of this phenotype, which should be taken into account when designing therapeutic strategies.

In our adjusted logistic regression model for D2TRA status, two variants—rs1813443 (*CNTN5*) and rs12081765 (intergenic, near *LMX1A*/*RXRG*)—showed nominal significance (*p* < 0.05). However, neither survived correction for multiple testing (minimum FDR-adjusted *p* = 0.49). The recurrence of rs1813443 as the lowest *p*-value across several analytical approaches is noteworthy but should not be overinterpreted; given the modest sample size and the number of tests performed, this observation is best described as a hypothesis-generating signal requiring independent validation in a larger, well-phenotyped cohort.

### 3.3. Why Do Candidate-Gene Studies in RA Consistently Fail to Replicate?

The failure of candidate-gene pharmacogenetic studies to yield robust, replicable findings in RA is multifactorial. First, sample size is a critical limitation. Most published candidate-gene studies, including the present one, are powered only to detect large genetic effects (OR ≥ 2.0 at MAF ≈ 0.3). However, the true effect sizes for common variants influencing treatment response are likely to be modest (OR 1.1–1.5). Detecting such small effects requires very large sample sizes—typically thousands of patients per phenotype—which are rarely available in single-center studies.

Second, phenotype definition varies widely across studies. Response to anti-TNF or anti-IL-6 therapy has been defined using different thresholds (ACR20/50/70, EULAR good/moderate response, DAS28 remission, etc.), at different time points (3, 6, or 12 months), and in different treatment settings (first-line versus later-line, with or without concomitant MTX). This lack of standardization substantially limits the comparability of results across cohorts and may contribute to the failure to replicate initial findings.

Third, population stratification and genetic heterogeneity across ethnic groups can confound association signals, particularly when candidate variants have different allele frequencies or effect sizes in different populations. Our study, conducted in a single-center cohort of predominantly Russian patients, may not be directly comparable to studies performed in Western European, North American, or Asian populations.

Fourth, treatment heterogeneity within drug classes—as exemplified by the drug-specific effect of rs1800629 for etanercept but not infliximab [12]—means that pooling patients receiving different agents within the same class may obscure a genuine association that is specific to one drug. In our anti-TNF phenotype, we aggregated patients receiving adalimumab, etanercept, golimumab, and certolizumab pegol; if a genetic effect is drug-specific, this aggregation would reduce statistical power and potentially mask a true association.

Fifth, clinical and demographic covariates—including age, sex, body mass index, disease duration, baseline disease activity, concomitant MTX or glucocorticoid use, smoking status, and comorbidities—are known to influence treatment response. Our unadjusted allelic screen for anti-TNF and anti-IL-6 response did not account for these factors, which may have further limited our ability to detect modest genetic effects.

Finally, the biological complexity of treatment response extends beyond germline genetic variation. Emerging evidence indicates that synovial tissue pathotypes, fibroblast-like synoviocyte (FLS) phenotypes, epigenetic modifications, and the composition of immune cell infiltrates in the joint may be as important—or more important—than host genetics in determining response to biologic therapy. For example, the pauci-immune synovial pathotype has been associated with inadequate response to TNF-α blockade [16]. In addition, demographic and lifestyle factors such as age, sex, and dietary intake can influence serum biomarker levels in the general population [26], and may similarly confound the interpretation of biomarker associations in RA. Furthermore, FLS can acquire an autonomous, aggressive phenotype driven by epigenetic reprogramming, sustaining inflammation independently of immune signals [27]. These non-genetic mechanisms may explain a substantial proportion of the variability in treatment response that cannot be captured by genotyping candidate variants alone.

### 3.4. Implications for Future Research

Genetic markers currently add limited incremental value beyond clinical and synovial predictors of treatment response. Synovial tissue pathotypes—particularly when assessed by RNA-seq rather than histopathology—predict differential response to specific biologic classes. In the R4RA biopsy-driven trial, a refractory subgroup characterized by failure of ≥3 biologic classes showed a signature of >1200 genes linked to the fibroid/pauci-immune pathotype [28,29], and in a b/tsDMARD inadequate responder cohort, patients with a non-inflammatory phenotype were twice as likely to have pauci-immune synovitis compared with the inflammatory group [19]. Stromal cell-mediated pathways, including fibroblast activation and extracellular matrix remodeling, may plausibly underlie treatment resistance independently of classical immune targets [18,19]. Clinical factors (baseline disease activity, disease duration, serology, prior treatment failures) remain universally available and form the foundation of all prediction models. Genetics nevertheless has practical advantages—stability over time, non-invasive assessment, and availability before any treatment exposure—and may eventually contribute as a complementary layer in combined models, particularly for pain-predominant D2TRA or drug-specific toxicity. At present, however, no genetic marker is recommended in EULAR or American College of Rheumatology (ACR) guidelines for routine prediction of biologic response, and a large community-based assessment questioned the utility of SNP information beyond standard clinical characteristics for predicting anti-TNF efficacy [19]. Even for MTX—the anchor drug in RA—a recent systematic review concluded that the available evidence remains predominantly of moderate quality, and current Clinical Pharmacogenetics Implementation Consortium (CPIC) and Dutch Pharmacogenetics Working Group (DPWG) guidelines do not recommend routine dose adjustments based solely on single gene variants [30].

Importantly, in our cohort, clinical and quality-of-life parameters differentiated D2TRA from non-D2TRA more strongly than any individual genetic marker, reinforcing the need for future studies to integrate genetic, clinical, and psychosocial data into unified stratification models rather than relying on candidate variants alone. This is consistent with recent real-world evidence: in a single-center cross-sectional study of 344 patients with established RA, D2TRA was associated primarily with clinical and serological factors—female sex, longer disease duration, higher RF and ACPA titers, elevated ESR, glucocorticoid use, and a greater number of failed advanced therapies—rather than with any molecular marker [31]. Notably, although both D2T and non-D2T groups achieved low disease activity or remission by DAS28 and CDAI, D2T patients were more frequently treated with JAKi (Filgotinib and Upadacitinib), which appeared associated with clinical stabilization [31].

A PRS approach was not feasible in the present study for three reasons: the panel comprised only 35 candidate variants; no adequately powered GWAS is currently available for anti-IL-6 response or D2TRA; and the sample sizes (66 for anti-TNF, 69 for anti-IL-6, 154 for D2TRA) are insufficient to validate a PRS without overfitting. The present study nonetheless establishes the targeted sequencing workflow and candidate variant set that can be extended to genome-wide discovery and PRS-based prediction once adequately powered, harmonized cohorts become available.

The findings of this study argue against the continued pursuit of isolated candidate-gene studies in small-to-moderate-sized RA cohorts [20]. Future research should prioritize adequately powered, multi-center cohorts with harmonized response definitions, followed by genome-wide analyses with rigorous replication rather than candidate-gene approaches. Given the observed heterogeneity of D2TRA, future studies should also stratify patients into biologically defined subtypes (e.g., PIRRA vs. NIRRA, or by synovial pathotype) to increase the power to detect subtype-specific associations.

### 3.5. Biological Plausibility of Nominally Associated Variants and Validation Strategy

The variants identified in this study require cautious interpretation. *MAP2K6* (rs11656130) encodes MKK6, a dual-specificity kinase that activates p38 MAPK, a pathway central to the production of TNF-α, IL-1β, and IL-6 in macrophages and synovial fibroblasts. A previous candidate-gene study of the p38 MAPK signalling network reported a nominal association between an intronic *MAP2K6* variant and anti-TNF response [32], but subsequent meta-analyses did not confirm this after correction [33]. In contrast, our cohort found rs11656130/MAP2K6 to be nominally associated with anti-IL-6 response, not anti-TNF response. *GALNT18* (rs4910008) encodes a glycosyltransferase involved in O-glycosylation; while not directly in the IL-6 pathway, it may influence IL-6R glycosylation or shedding. A GWAS of tocilizumab response identified *GALNT18* as a candidate locus [14], and a small candidate-gene study reported an association with EULAR response [34], but these findings require replication. *LINC02549* (rs7767069) is a long intergenic non-coding RNA; a large meta-analysis (*n* = 2067) reported an association with reduced DAS28 improvement after TNF inhibitor therapy, with functional correlates including altered memory T-cell subsets and soluble CD5/CD6 levels [35]. Our cohort showed a nominal association consistent with this direction but did not survive multiple-testing correction. *CNTN5* (rs1813443) encodes a neuronal cell adhesion molecule with limited known immune function. A large meta-analysis reported a weak, RF-specific association with reduced DAS28 improvement after TNF inhibitor therapy, but no functional correlates were identified, and the association did not survive multiple-testing correction [35]. The intergenic variant rs12081765 (annotated near *LMX1A*/*RXRG*) has been associated with anti-TNF response in the BRAGGSS cohort (*n* = 1828), although no direct functional data link it to RA drug response [36]. *RXRG* encodes a nuclear receptor of the RXR family, which has established immune-regulatory roles in macrophages and T cells [37], but whether this locus affects RA drug response through RXRG remains unknown.

To move beyond association, future work should prioritize replication in adequately powered, multi-center cohorts with harmonized response definitions; fine-mapping and eQTL/pQTL colocalization in immune and synovial cells; and functional assays, such as CRISPR editing or allele-specific reporter assays in macrophages or synovial fibroblasts, followed by cytokine measurements. For *LINC02549*, further characterization of its expression and regulatory targets in T cells and synovial fibroblasts is warranted. Until such validation is available, these variants should be considered hypothesis generating.

## 4. Materials and Methods

### 4.1. Patients and Phenotype Definitions

Patients with a confirmed diagnosis of RA were drawn from a single-center clinical database of 293 patients, comprising 99 MTX responders, 98 patients receiving b/tsDMARDs who did not fulfil D2TRA criteria, and 96 patients fulfilling D2TRA criteria. Three clinically defined binary phenotypes were evaluated.

(i) D2TRA status was assigned according to EULAR criteria, requiring: (a) failure of at least two b/tsDMARDs with different mechanisms of action following unsuccessful csDMARD therapy; (b) at least one sign of active or progressive disease, including moderate-to-high disease activity (DAS28-ESR > 3.2 or CDAI > 10), imaging or laboratory markers of active inflammation, inability to taper glucocorticoids below 7.5 mg/day prednisolone-equivalent, rapid radiographic progression, or well-controlled disease with persistent quality-of-life-limiting symptoms; and (c) perception of disease management as problematic by the treating rheumatologist and/or the patient. Patients receiving b/tsDMARD therapy who did not fulfill these criteria were classified as the non-D2TRA comparator group.
(ii) Response to anti-IL-6 therapy was defined as clinical response to one of tocilizumab, levilimab, olokizumab, or sarilumab.
(iii) Response to anti-TNF therapy was defined as clinical response to one of adalimumab, etanercept, golimumab, or certolizumab pegol.

Assessment of RA activity was based on the DAS28-CRP index (with CDAI and SDAI also available in the database). The therapeutic target was achievement of remission or low disease activity, defined as DAS28-CRP < 2.6—remission; DAS28-CRP 2.6–3.2—low disease activity. Response to therapy was defined as the achievement of low disease activity or remission (DAS28-CRP ≤ 3.2) at the last documented assessment while on the relevant therapy. Patients who discontinued therapy before 3 months due to inefficacy were classified as non-responders; those who discontinued due to adverse events were excluded from the response analysis, as their DAS28-CRP at the time of discontinuation may have been confounded by the adverse event itself. Of note, 32 patients received both anti-TNF and anti-IL-6 therapy: 28 patients were non-responders to both agents and therefore contributed to both non-responder groups, while 4 responded to both and contributed to both responder groups. The remaining 51 patients received other biologic classes (rituximab, abatacept, or JAKi) and were not included in the drug-specific analyses.

For the D2TRA phenotype, sample- and variant-level quality control was applied. Of the 258 patients for whom DNA was available, 99 belonged to the MTX-responder group and were not part of the biologic-treated analysis; 1 sample was excluded because it lacked a clinical record; 2 samples were excluded as technical duplicates with 100% genotype concordance (the lower-numbered identifier was retained in each pair); and 2 samples were excluded for implausible age values. After these exclusions, the primary analysis set comprised 154 patients (59 D2TRA and 95 non-D2TRA) with complete covariate data.

### 4.2. Targeted Panel Design and Library Preparation

Candidate variants were selected from the literature based on previously reported associations with treatment response, cohort size and characteristics, effect size, statistical significance, and biological plausibility of the gene’s involvement in the drug’s mechanism of action. The initial literature review identified 43 candidate variants from published pharmacogenetic studies of RA biologic therapy response. Two pairs of variants mapped to the same genomic position with different alternate alleles, yielding 41 unique positions. Primer pairs were designed for each position using NGS-PrimerPlex v.1.3.4 and PCR conditions were empirically optimized. Successful amplification was achieved for 39 of the 41 unique positions; two variants failed amplification and were dropped: rs113878252 (*MED15*) and rs10108210 (intergenic, chr8). The 39 successfully amplified variants were carried through hybridization capture, sequencing, and variant calling, yielding a final VCF of 39 variants. After exclusion of four variants for >20% missingness, 35 variants were tested in the association analysis. This set of 35 variants was used consistently for all three phenotypes (anti-TNF, anti-IL-6, D2TRA). A summary of the variant selection pipeline is provided in Supplementary Table S1. It should be noted that many published studies have focused on drug-target genes or RA susceptibility loci; although genome-wide and transcriptomic approaches have been proposed, they often require invasive tissue sampling, whereas our panel provides a non-invasive, targeted alternative suitable for blood-based genotyping.

Genomic DNA was extracted from whole-blood samples using an Auto-Pure 96 automated station and the MagPure Universal DNA Kit (Magen, Guangzhou, China). DNA quality and purity were assessed by spectrophotometry (A_260/280_ ratio). Genomic libraries were prepared using the Raissol DNA Library Prep Kit (Sesana LLC, Moscow, Russia).

For probe generation, the 39 target regions containing the selected SNPs were amplified using individually designed primer pairs. After amplification, end repair, A-tailing, and adapter ligation were performed using adapters annealed from custom oligonucleotides: BAC oligo1 (5′-CCATCTCATCCCTGCGTGTCGACTACACTACTCGT-3′) and BAC oligo2 (5′-[phos]CGAGTAGTGTTCAGCAAGGCACACAGGGGATAGG-3′). Subsequently, PCR with biotinylated primers—BAC-for-bio (5′-bio-CCATCTCATCCCTGCGTGTC-3′) and BAC-rev-bio (5′-bio-CCTATCCCCTGTGTGCCTTG-3′)—was carried out for six cycles to obtain sufficient biotinylated library (>1 µg). Products were purified using AMPure XP beads (Beckman Coulter Inc., Brea, CA, USA).

### 4.3. Hybridization Capture and Sequencing

Target enrichment was performed by hybridization capture using the biotinylated probe pool, with biotin–streptavidin capture on Dynabeads™ MyOne™ Streptavidin C1 magnetic beads (Thermo Fisher Scientific, Schwerte, Germany). Hybridization was carried out at 65 °C for 48 h in the presence of Cot-1 blocking DNA and relevant oligonucleotides. Captured libraries were washed with high- and low-stringency buffers at controlled temperatures, re-amplified on-bead, and purified with AMPure XP beads. Sequencing was performed on a DNBSEQ G400 (2 × 150 bp paired-end reads) (MGI, Shenzhen, China), targeting approximately 1 million paired reads per sample. The actual mean yield was 4.06 million total reads (median 3.97 million), corresponding to approximately 2.03 million paired reads per sample. Per-variant mean sequencing depth across all 258 samples ranged from 66× to 851×, with an overall mean of 571× and median of 605×. Two variants had relatively low mean depth: rs10919563/*PTPRC* (66×) and rs7767069/*LINC02549* (73×); both were retained in the analysis as their missingness in the 154-patient analysis set was 0%.

### 4.4. Variant Calling and Quality Control

Reads were aligned to the human reference genome (hg38/GRCh38) using BWA-MEM v.0.7.19-r1273 with default parameters [38]. Sequence variants were called with GATK HaplotypeCaller v.4.5.0 (BP_RESOLUTION mode) [39]. Genotypes were coded as dosage (0/1/2) of the first-listed alternate allele. The chrX variant (rs7055107) was coded with sex-specific dosage. Genetic association study was conducted using PLINK2 (v2.0.0-a.6.9) [40] with logistic regression models adjusted for age and sex.

Of the 39 variants in the VCF, 31 had 0% missingness in the 154-patient analysis set, 4 had low missingness (2.6–11.7%), and 4 exceeded the 20% missingness threshold and were excluded: rs1800629 (76.6%), rs361525 (76.6%), rs12083537/*IL6R* (27.3%), and rs1801274/*FCGR2A* (20.8%). The remaining 35 variants were tested. Notably, the two *TNF* promoter variants excluded for high missingness (rs1800629 and rs361525) showed a characteristic pattern of highly skewed depth distributions: mean depth of 588× and 605×, respectively, but median depth of only 73× and 76×. This reflects a batch effect—these variants were successfully sequenced at high depth in the first batch of approximately 60 samples but had near-zero coverage in the remaining approximately 198 samples, confirming that the high missingness is attributable to batch-specific amplification failure rather than globally poor coverage.

Hardy–Weinberg equilibrium (HWE) was tested in the non-D2TRA control group for each autosomal variant (the chrX variant was tested in females only) using a chi-square test. No variant showed deviation from HWE exceeding the *p* < 0.001 threshold. The lowest HWE *p*-values were 0.0187 (rs284511), 0.0209 (rs1568885), and 0.0243 (rs4240847), all well above the 0.001 threshold. HWE *p*-values for the four excluded variants could not be computed reliably due to the high proportion of missing genotypes.

Technical duplicate pairs were identified and resolved by genotype-concordance verification across assayed variants (100% concordance in all evaluable pairs); the lower-numbered sample identifier was retained in each pair. Samples lacking clinical or genotype data, or with implausible age values, were excluded. Per-variant sequencing depth, missingness, genotype counts, MAF, HWE *p*-values, and final inclusion/exclusion status for all 39 variants in the VCF are provided in Supplementary Table S1. Effect alleles were defined as the first ALT allele in each VCF record. All secondary ALT alleles at multi-allelic sites had MAF = 0 in this cohort and were not tested separately.

### 4.5. Statistical Analysis

All statistical analyses were performed using Python v.3.14.2. Continuous variables are presented as median [Q1; Q3] and compared using the Mann–Whitney U test. Categorical variables are presented as *n* (%) and compared using the χ^2^ test; when any expected cell count was <5, Fisher’s exact test was used instead. All tests were two-sided, and *p* < 0.05 was considered statistically significant. No correction for multiple comparisons was applied to the clinical and quality-of-life analyses, as these were considered descriptive and hypothesis generating.

For anti-IL6 and anti-TNF response, association between each genotyped variant and phenotype was tested using a one-degree-of-freedom chi-square allelic test (unadjusted screen; 35 variants tested per phenotype after exclusion of variants with >20% missingness). Multiple-testing correction was applied using Bonferroni and FDR (Benjamini–Hochberg, BH) methods across the tested variants within each phenotype. This unadjusted screen does not account for age, sex, body mass index, disease duration, baseline disease activity, or the specific bDMARD received within each drug class and should be interpreted as an exploratory allelic screen rather than a fully adjusted clinical-response model. A sensitivity analysis additionally adjusting for disease duration was performed; results were highly concordant with the primary model (Spearman ρ = 0.93 for anti-TNF, 0.85 for anti-IL-6).

For D2TRA status, the primary analysis was an additive logistic regression model (D2TRA status~genotype dosage + age + sex + body mass index) fitted in the 154-patient quality-controlled analysis set (Section 4.1), with dominant and recessive coding tested as secondary models and unadjusted Cochran–Armitage trend and Fisher’s exact tests as concordant sensitivity analyses; correction for the 35 tested variants used Benjamini–Hochberg FDR (primary) and Bonferroni (strict sensitivity, α/35 = 0.00143). All tests were two-sided; *p* < 0.05 was considered nominally significant prior to correction.

## 5. Conclusions

A literature-derived, 35-variant targeted sequencing panel, evaluated across three clinically defined phenotypes of biologic therapy response and D2T status in RA, did not yield any variant that retained statistical significance after correction for multiple testing. We report the candidate-selection rationale, targeted sequencing workflow, and bioinformatic/statistical pipeline in detail so that they can be reused directly for further work. These findings support prioritizing adequately powered, multi-center studies with harmonized treatment-response definitions and ancestry-aware analyses over further interpretation of isolated nominal associations from small candidate-gene cohorts.

### Limitation of Our Study

Several limitations of this study should be acknowledged. The sample size was modest for all three phenotypes. With the current sample sizes, the study had 80% power to detect only large genetic effects (OR ≥ 3.9 for D2TRA; OR ≥ 8.5–9.3 for anti-TNF and anti-IL-6 at MAF = 0.3, α = 0.05 uncorrected). At the Bonferroni-corrected threshold (α = 0.00143), the study was underpowered even for large effects (OR > 50 for anti-TNF and anti-IL-6; OR ≥ 8.0 for D2TRA). The study was not adequately powered to detect modest effects (OR 1.1–1.5), which are more typical for common variants influencing treatment response.

For the anti-TNF and anti-IL-6 phenotypes, the analyses were not adjusted for MTX use or line of treatment. Ever-use of MTX had near-zero variance, and concomitant MTX use was populated for only approximately half of relevant therapy episodes. Line of treatment is a consequence of the phenotype definition rather than a confounder, and adjusting for it would introduce collider bias. Disease duration was the only suitable covariate; a sensitivity analysis adjusting for it yielded highly concordant results.

The definition of response—achievement of low disease activity or remission (DAS28-CRP ≤ 3.2) at the time of assessment—while consistent with the therapeutic target, may not capture the full spectrum of treatment response, including late responses or drug-specific differences in response kinetics. Because the study used a cross-sectional cut of the RA registry, the duration of the last treatment stage varied across patients (minimum 3 months), and response was determined retrospectively rather than prospectively.

The anti-TNF and anti-IL-6 phenotypes each aggregate several pharmacologically distinct agents, with sequential switches made by a rheumatology commission based on clinical course, comorbidities, and infection risks. If genetic effects differ by specific drug, aggregating across agents could obscure a true association. This is the same source of heterogeneity widely cited as limiting the reproducibility of candidate-gene findings in RA, and it applies equally to our own negative results.

No independent validation cohort was available to confirm the nominal associations observed for rs1813443 and rs12081765. These should be considered exploratory findings requiring replication.

Population stratification could not be formally corrected using principal-component analysis, as the candidate panel of 35 variants does not provide a reliable genome-wide representation for ancestry inference. Other ancestry information (e.g., self-reported ethnicity) was not available for all participants. The elevated genomic inflation factor (λ_GC_ = 1.60) in the adjusted D2TRA model should be interpreted cautiously in this context and cannot be used to distinguish population stratification from small-sample variability.

The single-center design may limit generalizability, and the findings may not reflect the genetic and clinical heterogeneity of broader RA populations.

A potential limitation of the present study is the differential retention of patients between groups (95 of 98 non-D2TRA vs. 59 of 96 D2TRA from the original clinical database), resulting in an unbalanced final analysis set (95 vs. 59) that may introduce selection bias.

Finally, because the tested variants were selected from prior literature rather than from a genome-wide screen, the absence of significant association after correction indicates that these candidate variants did not show a robust association in this cohort; it does not indicate that no genetic predictors of bDMARD response exist.

## Figures and Tables

**Table 1:** Nominally significant (uncorrected *p* < 0.05) variants by phenotype, unadjusted allelic screen.

Phenotype	Variant	Unadjusted *p*	FDR (BH)	Bonferroni
anti-IL6	rs11656130/*MAP2K6*	0.0163	0.3704	0.5705
anti-IL6	rs4910008/*GALNT18*	0.0212	0.3704	0.7420
anti-TNF	rs7767069/LINC02549	0.0171	0.5968	0.5985

**Table 2:** Candidate genetic variants reported to be associated with RA therapy response, and their association with D2TRA (versus non-D2TRA) after adjustment for age, sex, and body mass index.

rsID	Gene	Chr	hg38	Drugs	Population (Based on Literature)	A1	A2	OR	95% CI	Add *p*	Add FDR	Dom *p*	Rec *p*
rs1061622	*TNFRSF1B*	1	12,192,898	anti-TNF	Italian Caucasian	G	T	0.99	[0.57, 1.74]	0.983	0.989	0.892	0.737
rs11265618	*IL6R*	1	154,457,616	anti-IL6	White Caucasian	T	C	0.64	[0.31, 1.30]	0.215	0.727	0.321	1.000
rs12081765	intergenic *LMX1A*/*RXRG*	1	165,372,612	anti-TNF	Caucasian origin	G	A	0.59	[0.37, 0.94]	0.028	0.490	0.050	0.094
rs4651370	*PLA2G4A*	1	187,269,960	anti-TNF	European origin	A	T	1.12	[0.53, 2.35]	0.773	0.962	0.504	N/A
rs12142623	*PLA2G4A*	1	187,321,274	anti-TNF	European origin	A	C	1.15	[0.55, 2.38]	0.710	0.962	0.453	N/A
rs10919563	*PTPRC*	1	198,731,313	anti-TNF	white European	A	G	1.20	[0.59, 2.45]	0.611	0.930	0.839	0.301
rs4240847	*MAPKAPK2*	1	206,723,277	anti-TNF	Caucasian	A	C	0.69	[0.43, 1.11]	0.122	0.727	0.255	0.149
rs6427528	*CD84*	1	160,546,518	anti-TNF	European/Portuguese	G	A	0.63	[0.31, 1.29]	0.207	0.727	N/A	0.372
rs10797077	*CD84*	1	160,545,069	anti-TNF	Portuguese	A	G	0.72	[0.34, 1.53]	0.393	0.727	1.000	0.528
rs703297	intergenic	2	19,426,057	anti-IL6	European	T	C	0.83	[0.49, 1.40]	0.477	0.794	0.496	0.624
rs39699	*EPHB1*	3	134,979,342	anti-IL6	white	G	A	0.76	[0.48, 1.20]	0.241	0.727	0.753	0.113
rs1447722	intergenic	3	139,835,611	anti-TNF	European origin	G	C	0.81	[0.48, 1.37]	0.431	0.754	0.718	0.290
rs1532269	*PDZD* *2*	5	32,018,735	anti-TNF	Caucasian origin	G	C	1.58	[0.97, 2.58]	0.068	0.727	0.297	0.063
rs703505	*KCNMB1*, *KCNIP1*	5	170,382,398	anti-IL6	European	G	A	0.70	[0.44, 1.10]	0.120	0.727	0.333	0.092
rs17301249	*EYA4*	6	133,291,776	anti-TNF	Caucasian origin	C	G	1.69	[0.83, 3.42]	0.147	0.727	0.130	0.795
rs916344	*MAPK14*	6	36,121,162	anti-TNF	Caucasian	G	C	0.84	[0.43, 1.63]	0.600	0.930	0.354	0.783
rs7767069	*LINC02549*	6	68,060,671	anti-TNF	European origin	A	T	0.95	[0.58, 1.54]	0.825	0.962	0.531	0.899
rs284511	*MAP3K7*	6	90,498,823	anti-TNF	Japanese	C	T	0.79	[0.51, 1.23]	0.299	0.727	0.959	0.082
rs1568885	intergenic	7	13,597,906	anti-TNF	European origin	A	T	1.04	[0.54, 1.98]	0.907	0.972	0.594	0.380
rs1813443	*CNTN5*	11	100,140,279	anti-TNF	European origin	C	G	1.85	[1.12, 3.06]	0.017	0.490	0.013	0.241
rs475032	*RPS6KA4*	11	64,373,265	anti-TNF	Caucasian	C	G	1.27	[0.78, 2.07]	0.337	0.727	0.606	0.320
rs4910008	*GALNT18*	11	11,458,319	anti-IL6	European	T	C	0.94	[0.58, 1.50]	0.782	0.962	0.893	0.754
rs717117	*LRRC55*	11	57,127,131	anti-TNF	European origin	G	A	0.69	[0.29, 1.62]	0.395	0.727	0.460	1.000
rs11052877	*CD69*	12	9,753,094	anti-IL6	European	G	A	0.75	[0.47, 1.20]	0.234	0.727	0.103	0.913
rs7305646	intergenic	12	17,111,403	anti-TNF	Caucasian origin	C	T	0.76	[0.48, 1.22]	0.254	0.727	0.318	0.380
rs1560011	*CLEC2D*	12	9,670,356	anti-IL6	European	G	A	1.06	[0.67, 1.68]	0.800	0.962	0.564	0.952
rs3794271	*SLCO1C1*	12	20,707,159	anti-TNF	Spanish	A	G	1.29	[0.81, 2.06]	0.284	0.727	0.811	0.165
rs9594987	*ENOX1*	13	43,656,858	anti-IL6	European	C	T	0.98	[0.62, 1.53]	0.916	0.972	0.998	0.860
rs2378945	*NUBPL*	14	31,831,584	anti-TNF	Spanish European	A	G	1.23	[0.78, 1.95]	0.372	0.727	0.196	0.958
rs1286076	*RPS6KA5*	14	90,980,083	anti-TNF	Caucasian	T	C	1.00	[0.58, 1.74]	0.989	0.989	0.165	0.506
rs1286112	*RPS6KA5*	14	90,946,417	anti-TNF	Caucasian	G	C	1.04	[0.62, 1.73]	0.886	0.972	0.063	0.209
rs2716191	*MAP2K6*	17	69,540,181	anti-TNF	Caucasian	C	T	0.93	[0.57, 1.49]	0.749	0.962	0.820	0.456
rs11656130	*MAP2K6*	17	69,404,305	anti-TNF	Caucasian	G	T	0.92	[0.54, 1.57]	0.769	0.962	0.648	0.989
rs4411591	*LINC01387*	18	6,550,118	anti-TNF	European origin	TT	CT	1.33	[0.71, 2.48]	0.373	0.727	0.311	0.880
rs7055107	*SLC9A7*	X	46,712,049	anti-IL6	European	T	G	0.75	[0.48, 1.15]	0.184	0.727	0.524	0.106

Notes: A1 is the effect allele; ORs are reported per additional copy of A1 (additive coding 0/1/2). OR > 1 indicates that A1 is a risk allele for D2TRA; OR < 1 indicates a protective effect. A2 is the reference allele. For rs4411591 (indel), A1 = TT (deletion of C relative to reference CT) and A2 = CT. For rs7055107 (chrX), males are hemizygous (coded 0/1), and females are diploid (coded 0/1/2); sex was included as a covariate. Dominant (Dom) and recessive (Rec) *p*-values are nominal (unadjusted for multiple testing) and are reported as secondary genetic models only.

## Data Availability

The original contributions presented in this study are included in the article/Supplementary Material. Further inquiries can be directed to the corresponding author.

## Supplementary Materials

The following supporting information can be downloaded at: https://www.mdpi.com/article/10.3390/ijms27198800/s1.
